# Developmental Dysplasia of the Hip: A Review of Etiopathogenesis, Risk Factors, and Genetic Aspects

**DOI:** 10.3390/medicina56040153

**Published:** 2020-03-31

**Authors:** Stefan Harsanyi, Radoslav Zamborsky, Lubica Krajciova, Milan Kokavec, Lubos Danisovic

**Affiliations:** 1 Faculty of Medicine, Institute of Medical Biology, Genetics and Clinical Genetics, Comenius University in Bratislava, 811-08 Bratislava, Slovakia; lubica.krajciova@fmed.uniba.sk (L.K.); lubos.danisovic@fmed.uniba.sk (L.D.); 2 Department of Orthopedics, Faculty of Medicine, Comenius University and National Institute of Children’s Diseases, 833-40 Bratislava, Slovakia; radozamborsky@gmail.com (R.Z.); kokavec@dfnsp.sk (M.K.)

**Keywords:** developmental dysplasia of the hip, DDH, genetics, epigenetics, risk factor

## Abstract

As one of the most frequent skeletal anomalies, developmental dysplasia of the hip (DDH) is characterized by a considerable range of pathology, from minor laxity of ligaments in the hip joint to complete luxation. Multifactorial etiology, of which the candidate genes have been studied the most, poses a challenge in understanding this disorder. Candidate gene association studies (CGASs) along with genome-wide association studies (GWASs) and genome-wide linkage analyses (GWLAs) have found numerous genes and loci with susceptible DDH association. Studies put major importance on candidate genes associated with the formation of connective tissue (COL1A1), osteogenesis (PAPPA2, GDF5), chondrogenesis (UQCC1, ASPN) and cell growth, proliferation and differentiation (TGFB1). Recent studies show that epigenetic factors, such as DNA methylation affect gene expression and therefore could play an important role in DDH pathogenesis. This paper reviews all existing risk factors affecting DDH incidence, along with candidate genes associated with genetic or epigenetic etiology of DDH in various studies.

## 1. Introduction

DDH is a developmental disorder that leads to various aberrations in the building structures of the hip joint, leading to abnormalities in the socket for the femoral head and laxity in the surrounding ligaments. It includes a wide range of morphological aberrations and their resulting functional disorders [1]. These disorders can be manifested only by a mild laxity in the capsule of the hip joint, or they can lead to early osteoarthritis (OA), secondary femur damage and movement problems. Complications are typical, especially in older age, but are not an exception even in youth and in worse cases lead to total hip arthroplasty (THA) at an early age. In clinical practice, this diagnosis in children during their individual phases of growth has a tendency to exhibit either improvement to a milder, even physiological state or to more severe pathology. Phenotypical heterogeneity and trouble reaching a clinical consensus for diagnostics in adults have led to the need for better and earlier diagnostic methods, which could only be achieved by genetic examination.

**Figure 1:**
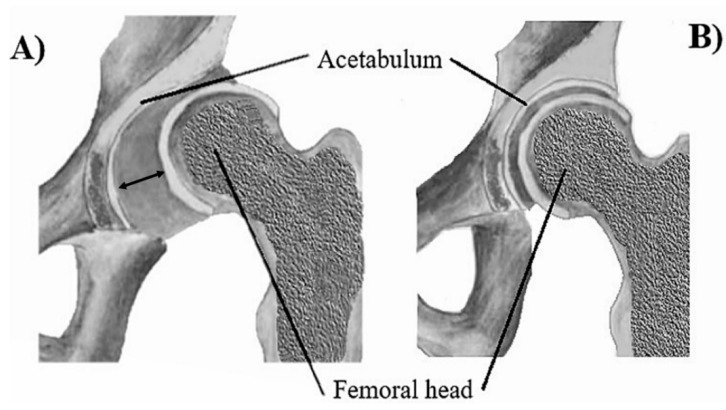
(**A**) Dysplastic acetabulum with a subluxated femoral head; (**B**) Acetabulum in physiological position.

Although isolated DDH can be diagnosed in healthy individuals, there are cases when extensive genetic mutations cause teratologic or syndromic DDH [2,3], which occurs prenatally. In syndromic types, DDH can be a part of many or only a sole manifestation of skeletal dysplasia [4,5,6], or it may be present in conjunction with other malformations, e.g., pes equinovarus and acetabular labrum abnormalities. Syndromic dysplasia exists also in association with different pathologies such as Down’s syndrome and neurogenic [7], renal or cardiovascular abnormalities. Nonsyndromic DDH is diagnosed as an isolated condition and its genetic component is targeted by scientists. Studies conducted on families with multiple individuals diagnosed with DDH have identified different chromosome loci that are associated with the occurrence of this disorder. These are mainly variants of genes whose products are structural factors of connective tissue, genes involved in osteo- and chondrogenesis, genes associated with the formation of joint structures and genes for chemokine receptors. Figure 1. presents the position of the femur and femoral head for a subluxated hip joint affected by DDH and a hip joint in physiological position, respectively.

## 2. Etiopathogenesis

Despite the long history of this disorder and the many specialists involved in this topic, there is still little knowledge about the exact etiopathogenesis of DDH. This is mainly due to the genetic, mechanical and environmental risk factors which define the multifactorial etiopathogenesis of DDH. The hip joint begins to develop physiologically from mesenchymal cells as early as the fifth or sixth gestational week. By the eleventh week, the femoral head is completely formed [8], and in the coming weeks it undergoes a more rapid growth than the acetabular cartilage, causing about 50% of the femoral cartilage to be present at the time of birth, but in the postnatal period, the cartilage begins to develop much faster. If laxity of the femoral head is present after birth, Neonatal Hip Instability (NHI) can be diagnosed. This instability is usually present for the first few weeks of life, has a mild course and up to 88% of cases of NHIs have a spontaneous resolution by the eighth week of life [9].

Persistent joint instability can be caused by a disorder of reflex contraction in soft tissues, which, under physiological circumstances, fixates the hip joint until six months of age. After the sixth month, spontaneous resolution is very unlikely [1] and the child needs intervention. Persistent laxity of the capsule, subluxation and/or dislocation lead to progressive dysplastic changes which, without therapy, can cause lasting consequences, especially in the case of a persistent dysplasia until adulthood. Persistent dysplasia over time causes alterations in body position and gait [10]. Reports of unilateral dysplasia are more severe than bilateral because the muscle strength in the affected limb is gradually affected [11]. Over time, the limb deforms in the form of shortening, either from cartilage reduction or other degenerative joint disease, and thus results in postural scoliosis, back pain and gradual disability of the patient.

## 3. Epidemiology

Ultrasound screening and monitoring of families with DDH history contributed to early DDH diagnosis and therefore earlier treatment, which over time led to a rapid decrease in THAs [12]. In case of late diagnosis, chances of long-term complications rise exponentially and this fact leads to a higher possibility of needing surgery [13]. Although the diagnosis of DDH is characteristic for an early age, diagnoses in adolescence or adulthood are not unusual [14].

According to literature, DDH incidence in newborns considerably varies with geographical location from lowest in Africans, to highest in Native Americans and Caucasian population. The global incidence can roughly be estimated to 0.1–6.6 cases per 1000 live births and is responsible for up to 30% of primary THAs in people up to 60 years of age [15,16,17,18,19]. Variations in incidence considering geographical location can be caused by epigenetic factors, consanguinity rates or study limitations, e.g., group size and diagnostic methods used. Compared to the general population in families with DDH history, the incidence was increased sevenfold between siblings and tenfold in the parents of probands. The concordance rate frequency (identical pathology in twins) was 33–41% for identical twins (monozygotic) and 3–8% for dizygotic twins [20]. Since there is more knowledge available about this topic, various countries have reported improvement in DDH incidence over the years [12,21].

Although we do not know the exact etiology of DDH, we know of risk factors, that contribute to the incidence of primary or secondary hip dysplasia. Table 1. presents these risk factors (genetic, epigenetic, mechanical and other) that include a number of areas that are not statistically significant but may be of clinical importance nevertheless [22].

## 4. Genetic Aspects

Although every patient with DDH needs to be assessed individually by precise radiological and clinical examination, extensive knowledge of the genetic background would greatly contribute to the diagnostic and therapeutic process. Factors such as specific screening programs for children with positive family history and gene-based prediction of disease behavior and severity could significantly influence the management of these patients.

A family in Utah had an inheritance pattern in four generations consistent with an autosomal type of inheritance with variable expressivity [42]. This type of DDH with a suspected mutation in the CX3CR1 gene has been designated type 2. DDH type 1 is characterized by a multifactorial type of inheritance and a variable phenotype with a number of candidate genes, many of which are investigated by various studies. The long arm of the 17th chromosome contains a region of HOX genes that are significantly associated with DDH. This region is essential for mesenchymal cell positional information in developing joints [43].

For a long time, it was believed that individual candidate genes are responsible for certain malfunctions in the physiology of connective tissue or structural proteins of the joint capsule and by this mechanism cause DDH. A two-gene system in DDH has been proposed by some authors, where one gene is responsible for joint laxity and the second affects e.g. acetabular abnormalities [44]. However, to create a pathology of this complexity, the background of DDH cannot be caused by a single or double gene aberration. It is more likely that the multifactorial etiology is affected by a larger number of genes and thus proving this disorder to be polygenetic.

The development of new, more advanced methods in molecular biology and genetics brought a significant change to molecular diagnostics. However, NGS (next-generation sequencing) is yet to be widely implemented in genetic research. Study designs used to identify the majority of associated genes in DDH research are CGASs (candidate gene association studies) along with genome-wide association studies (GWASs or WGASs—whole-genome association studies). RFLP (Restriction fragment length polymorphism) is an older method, that was used to identify single-nucleotide polymorphisms (SNPs) and polymorphisms. Nowadays, RFLP is not preferred for its low output. On the other hand, WES (whole-exome sequencing), an NGS method with a much larger output, is becoming the focus in genetic studies. As of yet, only three genes have been identified by WES: the CXCR1 (C-X-C motif chemokine receptor 1) gene in 2013, the BMS1 (ribosome biogenesis factor) gene in 2018 [45] and the TENM3 (teneurin 3) gene in 2019 [46].

CGAS utilizes a single-gene approach that offers certain advantages for the most relevant genes, which are prioritized [47,48]. We chose these genes based on a hypothesis about a specific role of a gene, or multiple pathway-related genes, on a certain phenotype. There are two primary approaches to choosing a candidate gene. First, a candidate gene is chosen based on the presence of a large effect mutation in a specific gene that potentiates more severe phenotypes of the studied disease. The second approach to identify a candidate gene is to choose a gene based on a biochemical process known to be associated with the etiology of the studied disease. Structures of these studies are usually case-control, in which pathological cases of the studied disease and healthy controls are first identified, and later examined for SNPs, haplotypes and copy-number variants (CNVs).

GWAS is a systematic observational study of a genome-wide set of genetic variants in individuals, which are based either on genotype or phenotype for the studied trait. The focus of these studies is on SNPs, which are characterized by the substitution of a single nucleotide in a specific position and therefore create variants or alterations of genes [49]. GWAS utilizes microsatellites and SNP tags. In the past, even though they were able to identify new genes and regions previously not associated with a disease, the efficacy of GWASs was in question, because using microsatellites yielded low output and frequent false positivity, which was slowly diminishing the importance of this method. However, NGS and advancement in genotyping systems brought SNP-based GWASs that are able to use more than a million markers at once, which have proven very effective in identifying regions important in the development of autoimmune and genetic disorders. In fields of study such as psychiatry, association studies have proven much more effective, than CGASs [50].

Systematic studies using another approach in genetic diagnostics are genome-wide linkage analyses (GWLAs), which utilize pedigrees to identify loci and their effects characteristic for a certain disease. Linkage analyses are also used for genetic mapping in cases where the evaluation of genetic predisposition is essential. The multifactorial nature of inheritance in DDH is a problematic aspect, as the presence of a potentially pathological genetic finding may not have a phenotypic manifestation. Phenotypic variability in families has been observed [51].

**Figure 2:**
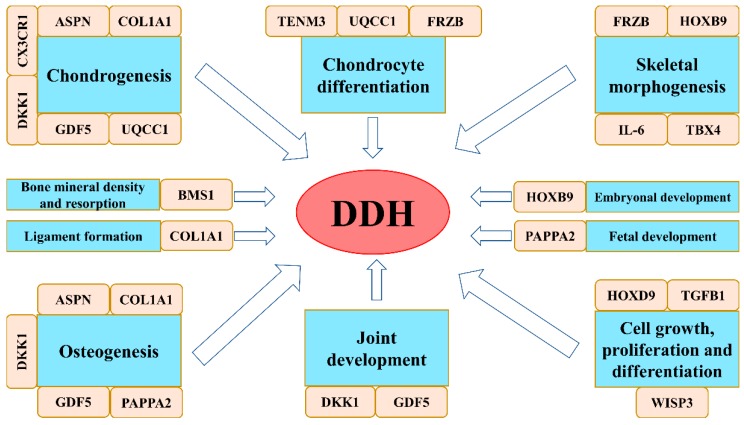
Genes associated with DDH and their suspected effects.

Studies in Europe indicate that the main genes involved in DDH pathology are genes for IL-6 (interleukin 6) [52], TGFB1 (transforming growth factor beta 1 or TGF-β1) [52] and GDF5 (growth differentiation factor 5 or cartilage-derived morphogenetic protein 1) [49]. Although frequently studied in the Chinese population, PAPPA2 (pappalysin 2 or pregnancy-associated plasma protein-A2) needs yet to be thoroughly studied in the European population [53]. Table 2. presents an up-to -date list of genes positively associated with DDH in literature along with their specific localization, SNPs, study designs and original publication. Figure 2. presents these associated genes and their suspected effects in the pathogenesis of DDH.

IL-6 is linked to DDH with its complex role in the physiology of bone metabolism, calcium and vitamin D levels, which plays a considerable role in the pathogenesis of osteoporosis [63]. Mutations of a single nucleotide—SNPs—located in the promoter of IL-6 are linked to OA [64], which is often the result of an untreated DDH. A symptomatic distal interphalangeal (DIP) osteoarthritis can also be caused by these mutations, which affect IL-6 secretion and transcription [65]. Lower susceptibility to OA along with lesser radiological impairment in both knee and hip joints has been observed in individuals carrying the C allele. If compared to the GG genotype, a reduction in IL-6 serum levels was observed in individuals with CC and CG genotypes. The presence of a G allele at the IL-6 polymorphic promoter in patients with DIP OA was associated with severe symptomatic and symmetrical outcomes.

TGF-β1 is a pro-inflammatory cytokine that plays a role in the etiopathogenesis of OA. TGF-β1 plays a crucial role in the regulation of perichondrial and fibroblast cells in tendons, in bone remodeling process, and in the regulation of cartilage development [66,67]. Control of cell growth along with differentiation, proliferation, and apoptosis are the main functions of this protein [68]. Polymorphisms in the TGFB1 gene are significantly associated with OA of the hip joint in studies based on the Croatian population [52].

GDF5 is a member of TGF-beta superfamily [69] and plays a key role in the formation of bones and synovial joints, endochondral ossification, maintenance of tendons and other musculoskeletal processes [70]. A broad spectrum of skeletal disorders and abnormal development of joints have been associated with aberrations in the human GDF5 gene [71,72]. Association between GDF5 and OA has been presented by several studies [73]. The risk allele T causes a reduction of expression of the GDF5 promoter sequence activity and increased risk for OA [74], which has also been observed by Rouault et al. in the Caucasian population [75].

PAPPA2 is a bone formation stimulator. Studies indicate, that involvement in normal postnatal growth may also be a function of this gene [76]. Osteoblasts during fetal development and differentiating chondrocytes during the endochondral bone formation express the substrate for PAPPA2 [53]. PAPPA2 encodes a novel metalloproteinase pappalysin 2 which may play roles in fetal development [77,78]. An animal model study on mice showed the possibility of pappalysin 2 involvement in DDH etiology by the interference in the cartilaginous and fibrous metabolism via proteins associated with the IGF signaling pathway [79].

## 5. Epigenetics

Epigenetic modifications cause variations in gene expression. In this case, heritable alterations in the expression of genes that result in a variation of the phenotype do not affect the DNA sequence, thus changes to the genotype are not involved. Several factors including age, environment, and specific disease state are the main influence that potentiates the epigenetic changes [80].

First of the epigenetic modifications is DNA methylation, which has a profound effect on gene expression and thus on the development of diseases associated with them. DNA methylation has an inhibitory effect on gene expression. The addition of methyl groups (-CH_3_) is controlled in cells and carried out by DNMT (DNA-methyltransferase) enzymes. These enzymes transport the methyl group (-CH_3_) to cytosine residues to form 5-methylcytosine, which exists in a CpG (cytosine-guanine) sequence. DNA methylation leads to chromatin remodeling and gene suppression by two mechanisms: directly mediated inhibition of DNA binding transcription factors and the other is mediated by specific proteins that contain a methylated DNA binding domain that binds between histones and methylated DNA [81,82]. A recent study found that the condition of methylation of the GDF5 gene is dysregulated in DDH patients. This fact may cause changes in GDF5 expression. Since the GDF5 plays a very important role in the development of cartilages and bones, then a reduction in expression may be causative to the development of DDH [83].

A recent study comparing cartilages from three different groups of patients: a healthy 14-year old adolescent, patients with old-age arthritis and a 23-year-old young adult with OA after failed DDH treatment. This study showed, that the loss of DNA methylation at certain CpG sites in the promoter regions was associated with the expression of proteolytic enzymes in the chondrocytes of this patient. This loss of DNA methylation is also observed in primary old-age OA, which suggests that age is not the only denominator for the epigenetic “unsilencing” specific for OA [84].

The second epigenetic modification is histone modification, which usually is a post-translational modification of histones. The third epigenetic mechanism is non-coding RNA-associated gene silencing. A study in China analyzed the expression level of Col2A1 and found that it was significantly downregulated in DDH. Further, the expression of lncRNA H19 in the DDH showed that within the control and the study group, the expression of H19 was considerably decreased in the group of DDH patients [85]. An important transcription factor for chondrogenesis during skeletal development is SOX9, expressed by mesenchymal stem cells. If downregulated by histone modification and DNA methylation, SOX9 decreases the expression of collagen and aggrecan in chondrocytes [86,87].

## 6. Conclusions

Despite the existence of guidelines for evaluation, diagnostics and referral for DDH, authors find it troublesome to reach a consensus on diagnostics in adult patients. Evidence based medicine still lacks knowledge and more specific information about precise genetic examination and screening. This is also burdened by the coexistence of other syndromic or non-syndromic conditions in which hip dysplasia is only a manifestation. In clinical practice, individual clinical and radiological phenotypical characterization of each patient is essential. To resolve the heritability and find a better preventative strategy, gene variations need to be studied and correlated with physical examination. One possibility to achieve this goal is by a wide genetic screening program aimed at newborns with positive family history, or if spontaneous hip reduction within the first three months of life is not experienced. However, the collection samples for various diseases could prove very difficult, time-consuming and challenging.

## Figures and Tables

**Table 1:** Risk factors associated with developmental dysplasia of the hip (DDH).

Risk Factor	Reported Associations
Female gender	In literature female gender shows seven to nine times more frequent DDH diagnosis at birth, than male newborns [19,23].
Left hip joint	Isolated right hip dysplasia is the least common type. The most common is the affection of the left hip due to fetal positions where the left hip is leaning towards the spine of the mother [24], bilateral affection is also common [25,26].
Gravidity	Post-terminal gravidity increases the chance of DDH [19].
Delivery	There is a significant risk of DDH due to the high strain on lower extremities posed by the breech presentation [27], although breech babies are more likely to experience spontaneous resolution [22,28]. Vaginal delivery, although having other benefits, compared to cesarean section significantly increases the statistical risk for DDH [29].
Limited fetal mobility	Factors such as oligohydramnios [30], high birth weight (HBW) or primiparity [18], present an increased risk for DDH.
Swaddling	Certain populations have reported higher incidence rates of DDH due to tight swaddling techniques [31,32].
Family history	Family history and its genetic contribution increase twelvefold the risk of DDH incidence in first-degree relatives [33,34]. Caucasian population: IL-6, TGFB1 Asian population: COL2A1, DKK1, HOXB9, HOXD9, WISP3 Non-specific: COL1A1, CX3CR1, GDF-5, PAPPA2
Presence of different malformations	Down’s syndrome or foot malformations e.g. clubfoot [35,36]. Torticollis, metatarsus adductus, laxity of tendons.
Intra- or extra-articular instability of the joint	Usually consequences of poor care and/or injuries causing damage to the hip joint, e.g. inflammation of surrounding tendons, some diseases (Legg–Calvé–Perthes Disease), acetabular labrum lesions or abnormalities [37,38,39]. Ligamentous laxity leading to instability. In a recent study, hypertrophy of the Transverse Acetabular Ligament did not prove to be causative in DDH [40].
Altitude	A recent study in Tibet found increased DDH incidence with increasing altitude [41].

**Table 2:** Genes associated with DDH.

Gene	Localization	Reference SNP	Study Design	Original Publication
ASPN	9q22.31	D repeat polymorphism	CGAS	Shi, D. et al., 2011 [47]
BMS1	10q11.21	rs201298233	WES	Zhu, L. et al., 2019 [45]
COL1A1	17q21.33	rs113647555	CGAS	Zhao, L et al., 2013 [54]
CX3CR1	3q22.2	rs3732378	GWLA	Feldman, G.J. et al., 2013 [42]
		rs3732379	GWLA	Basit, S. et al., 2018 [55]
DKK1	10q21.1	rs1569198	CGAS	Liu, S. et al., 2014 [56]
FRZB	2q32.1	rs288326	GWAS	Evangelou E. et al., 2009 [57]
GDF5	20q11.22	rs143383	CGAS	Dai, J. et al., 2008 [58]
		rs143384	GWAS	Hatzikotoulas, K. et al., 2018 [49]
HOXB9	17q21.32	rs2303486, rs8844	CGAS	Hao, Z. et al., 2014 [59]
HOXD9	2q31.1	rs711819	CGAS	Tian, W. et al., 2012 [60]
IL-6	7p15.3	rs1800796	RFLP	Kolundžić, R. et al., 2011 [52]
PAPPA2	1q25.2	rs726252	CGAS	Jia, J. et al., 2012 [53]
TBX4	17q23.2	rss3744448	CGAS	Wang, K. et al., 2010 [61]
TENM3	4q34.3-q35.1	rs183721398	WES	Feldman, G.J. et al., 2019 [46]
TGFB1	19q13.2	rs1800470	RFLP	Kolundžić, R. et al., 2011 [52]
UQCC1	20q11.22	rs6060373	GWAS	Sun, Y. et al., 2015 [62]
WISP3	6q21	rs1230345	CGAS	Zhang, J. et al., 2018 [48]

CGAS—Candidate gene association studies; WES—Whole-exome sequencing; GWLA—Genome-wide linkage analysis; GWAS—Genome-wide association analysis; RFLP—Restriction fragment length polymorphism.
